# Thermal Studies of Nanoporous Si Films with Pitches on the Order of 100 nm —Comparison between Different Pore-Drilling Techniques

**DOI:** 10.1038/s41598-018-26872-w

**Published:** 2018-06-13

**Authors:** Qing Hao, Dongchao Xu, Hongbo Zhao, Yue Xiao, Fabian Javier Medina

**Affiliations:** Aerospace & Mechanical Engineering, University of Arizona, 1130 N Mountain Ave, Tucson, AZ 85721 USA

## Abstract

In recent years, nanoporous Si films have been widely studied for thermoelectric applications due to the low cost and earth abundance of Si. Despite many encouraging results, inconsistency still exists among experimental and theoretical studies of reduced lattice thermal conductivity for varied nanoporous patterns. In addition, divergence can also be found among reported data, due to the difference in sample preparation and measurement setups. In this work, systematic measurements are carried out on nanoporous Si thin films with pore pitches on the order of 100 nm, where pores are drilled either by dry etching or a focused ion beam. In addition to thermal conductivity measurements, the specific heat of the nanoporous films is simultaneously measured and agrees with the estimation using bulk values, indicating a negligible change in the phonon dispersion. Without considering coherent phonon transport, the measured thermal conductivity values agree with predictions by frequency-dependent phonon Monte Carlo simulations assuming diffusive pore-edge phonon scattering. In Monte Carlo simulations, an expanded effective pore diameter is used to account for the amorphization and oxidation on real pore edges.

## Introduction

At the nanoscale, phonon transport can be largely suppressed by phonon scattering at nanostructured boundaries or interfaces. A better understanding of phonon scattering by boundaries and interfaces is critical to many nanotechnology applications, such as thermal management of high-power electronics^1^, thermoelectric (TE) materials for power generation and refrigeration^2–4^, phonon focusing^5^, and thermal insulation^6^. As one example, micro- and nano-porous TE Si thin films were intensively studied in the past two decades due to the low price and earth abundance of Si^6–8^. A good TE material should have a high electrical conductivity σ, a high Seebeck coefficient *S*, but a low thermal conductivity *k*^9^. By introducing nanopores, high TE performance was achieved in thin films with bulk-like electrical properties but dramatically reduced lattice part of the thermal conductivity (*k*_*L*_)^10,11^.

In the literature, special attention has been paid to the large discrepancy in the reported *k*_*L*_ values of periodic porous Si films. In experiments, the nano- or micro-pores were often drilled with dry etching, i.e., reactive ion etching (RIE)^5,10,12–17^ or deep reactive ion etching (DRIE)^11,18–20^. Pores with >100 nm diameters were also drilled with a focused ion beam (FIB)^21^. Besides some studies^5,13–20^, the in-plane *k*_*L*_ at room temperature was much lower than theoretical predictions that assumed bulk-like phonon transport and diffusive phonon scattering by pore edges^22,23^. Further considering the diffusive phonon scattering by the top and bottom surfaces of a thin film did not largely reduce the divergence for ~100 nm or shorter pitches.

In one hypothesis, the observed *k*_*L*_ reduction has been attributed to the coherent phonon transport within the periodic nanoporous structure^10,15,24,25^, which can modify the phonon dispersion and thus lower the *k*_*L*_. At 300 K, such “phononic effects” can be critical to superlattices with atomically smooth interfaces and <5 nm periods^26^. However, the studied nanoporous thin films usually have a structure feature size of ~10 nm to micrometers, which is much larger than the dominant phonon wavelength for Si (1–10 nm at 300 K^27,28^). In addition, the usually rough pore edges should diffusively scatter phonons so that the phonon phase coherence is destroyed. The phononic effects are anticipated to be weak in this case. More recent comparison studies between periodic and aperiodic nanoporous films further suggested negligible phononic effects above 10 K for films with 300 nm pitches^16^, and above 14 K for Si nanomeshes with>100 nm pitches^19^. This conclusion was also supported by measurements on Si nanoporous films with 200–300 nm pitches, where incoherent phonon transport was confirmed at 300–1000 K^29^.

Only considering incoherent phonon transport, calculations for nanoporous films^27^ agreed well with the measurements by El-Kady *et al*. for nanoporous films with 500–900 nm pitches, 7–38% porosity, and 500 nm film thickness^30^. Similarly, agreement with theoretical modeling was also found in recent cross-plane *k* measurements on nanoporous In_0.1_Ga_0.9_N films with 450–900 nm pitches and a fixed 300 nm pore diameter^31^. This contradicted earlier cross-plane thermal measurements on nanoporous Si films with sub-micron feature sizes, where phononic effects were proposed to explain the *k*_*L*_ reduction^24^. For even smaller features, the measurement data of nanoporous films with a ~34 nm pitch^10^ were successfully explained with a slightly expanded effective pore diameter to account for the pore-edge amorphization and oxidation^28^. These pore-edge defects were emphasized in molecular dynamics simulations^32,33^ and were found in the transmission electron microscopy (TEM)^11^ or scanning electron microscopy (SEM)^16,17^ studies of real samples. As one example to show the influence of fabrication-introduced surface defects on phonon transport, *k* of RIE-patterned Si nanowires^34^ was far lower than that for Si nanowires synthesized by the vapor-liquid-solid method^35^.

For phonon transport analysis of nanoporous thin films, different bulk phonon mean free paths (MFPs) and phonon dispersion employed may lead to some divergence. On the other side, accuracy of some cited measurements should also be questioned. In some studies, a thin-film sample was transferred onto a micro-device for measurements. The possibly large thermal contact resistance between a thin film and the microdevice may overshadow the thermal resistance of the film itself and lead to large underestimation for reported *k* value^10,11^. Some unphysical fluctuations were found in temperature-dependent *k* values, indicating possible variation of the sample-device thermal contact during thermal measurements^10,11^. Furthermore, some distortion and damage of a fragile nanoporous film during the film transfer and the following fabrication processes may also strongly affect *k*_*L*_. Such issues were addressed in more recent studies using an integrated device fabricated from the same Si film or using micro time-domain thermoreflectance measurements on a suspended sample, where the measured *k* values were mostly comparable to or higher than the theoretical predictions at 300 K^5,13–16,18–20^.

As another important aspect of thermal studies, the nanopore-drilling processes are less emphasized for their impact on pore-edge defects. In practice, the pore-edge defects can vary significantly when different etching gases and conditions for RIE or DRIE are used. Increased pore-edge defects are anticipated for FIB drilling with typically more surface damages^36^. Therefore, it is hard to directly compare results by different researchers due to the sample variation. To address this issue, this work aims to better understand the impact of the selected nanopore-drilling technique on the resulting *k*_*L*_. Systematic thermal studies were carried out on nanoporous Si thin films with pitches on the order of 100 nm, where nanopores were drilled with a FIB or DRIE. With more pore-edge defects, nanoporous films patterned by a FIB consistently showed a lower in-plane *k* comparing with those for films drilled by DRIE. To justify the phonon dispersion modification, the volumetric specific heat *C* of the nanoporous thin films was also measured. In existing studies, the measured *k* values could be affected by both phononic effects and pore-edge defects, which were hard to be distinguished. In contrast, *C* should only depend on the phonon dispersion and can be used to justify the existence of phononic effects. For studied nanoporous thin films, temperature-dependent *C* values were consistent with the estimation using bulk-material *C* values, indicating negligible variation of the phonon dispersion. The measured *k* values also agreed well with predictions by phonon Monte Carlo (MC) simulations that used an effective pore diameter considering pore-edge defects revealed by electron microscopy studies. Coherent phonon transport was not required to explain the observed *k* reduction. The results from this work provided important guidance for phonon engineering in nanoporous materials.

## Results

### Device fabrication

**Figure 1:**
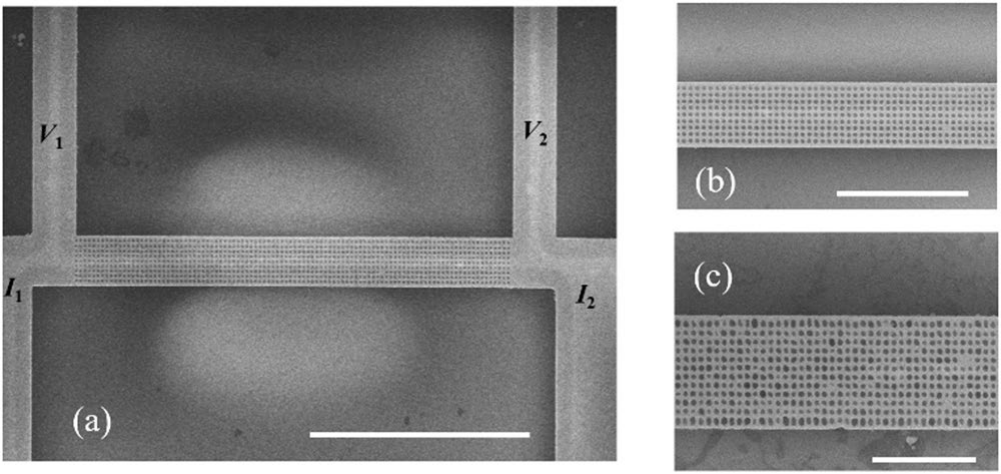
The SEM images of (**a**) suspended nanoporous Si film (diameter/pitch as 100/200 nm) with four electrical probes for thermal measurements and (**b**) the nanopores on this film. (**c**) The SEM image of the film with irregular pores over-etched from 50 nm to 94 nm in the average diameter. Scale bars are 10, 4, and 2 µm from (**a**) to (**c**), respectively.

The measured suspended film was fabricated from the 220-nm-thick device layer of a silicon-on-insulator (SOI) wafer. The nanoporous film and the four-probe electrical probes to the film were defined by electron beam lithography (EBL) and then etched by DRIE, as shown by the scanning electron microscope (SEM) image in Fig. 1a. The measured nanoporous film was 20 μm in length and 2 μm in width. The diameter/pitch combinations of aligned nanopores included 300/600, 200/400, 100/200, and 50/150 nm. The three larger porous patterns showed well-defined pore shape and generally <5 nm uncertainties in pore diameters (Fig. 1b). However, the smallest 50-nm-diameter pores became irregular after etching and the averaged porosity $\Phi$ was estimated as 31% using software (ImageJ) to read the SEM image (Fig. 1c). The effective pore diameter was estimated as 94 nm. The porous film was fully suspended by etching off the underneath oxide layer. Because the employed Si layer has a very low electrical conductivity (5–10 S/m), the whole structure was further coated with a 10-nm-thick Cr adhesion layer and then a 40-nm-thick Pt layer. The metallic coating was used as both heater and electrical-resistance thermometer in thermal studies, whereas current leakage through the Si film was neglected. In comparison, similar nanoporous films were also fabricated using a FIB for the 300/600 nm and 200/400 nm patterns. Instead, 10-nm-thick Cr and 20-nm-thick Au layers were coated onto FIB samples. Unlike DRIE drilling, even smaller pores could not be fabricated due to the limited aspect ratio for FIB drilling.

### 3*ω* measurements to extract both ***C*** and *k* of a nanoporous film

The challenge in the thermal studies of nanoporous thin films lies in that *k*_*L*_ can be affected by both the phonon dispersion modification and the scattering by amorphous pore edges. These two effects are difficult to be distinguished in the *k*_*L*_ analysis. In this work, this issue is addressed with specific heat measurements on a suspended nanoporous Si film, in addition to its in-plane *k* measurements. In physics, *C* is solely dependent on the phonon dispersion, whereas *k*_*L*_ also relies on the phonon scattering. The *C* and *k*_*L*_ measurements shown here can be applied to general nanoporous Si films to better understand the influence of amorphous pore edges and phononic effect on their *k*_*L*_.

**Figure 2:**
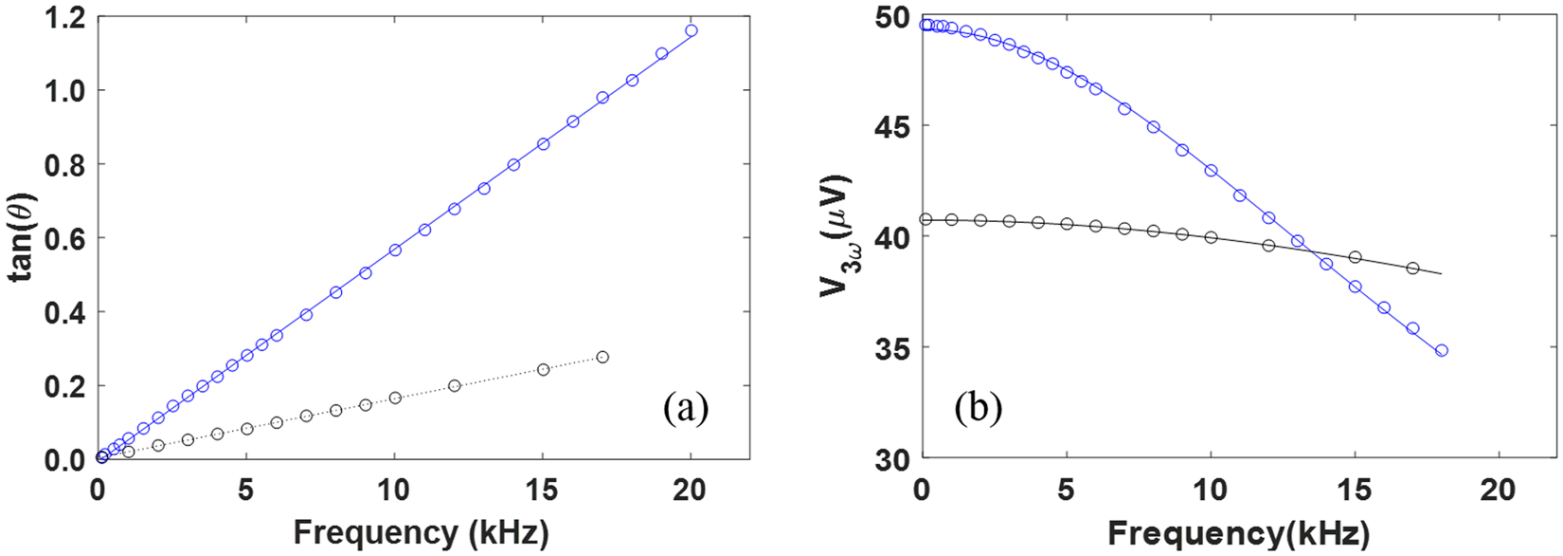
Frequency dependence of (**a**) $\tan (\theta )$ and (**b**) ${V}_{3\omega }$ in a typical $3\omega$ measurement for a nanoporous film (blue) and a solid film (black). Here symbols are measurement data and lines are for fitting.

Both the in-plane $k\approx {k}_{L}$ and volumetric specific heat *C* of a nanoporous film can be extracted from 3*ω* measurements developed for suspended samples^37^ (see Supplementary Information). The measured specific heat per unit volume, *C*, is divided by $1-\Phi$ to obtain the *C* value for the corresponding solid film. Figure 2a and b show fitting for frequency-dependent $\tan (\theta )$ and *V*_3*ω*_ functions at room temperature, where *θ* and *V*_3*ω*_ are the phase angle and root-mean-square voltage value of the 3*ω* signal, respectively. The black circles and fitting are for a solid film, whereas the blue circles and fitting are for a nanoporous film. Due to the higher thermal diffusivity *α* and thus reduced time constant *γ* for the suspended device, negligible *V*_3*ω*_ variation in Fig. 2b is observed for a solid film. At reduced temperatures, *γ* also becomes smaller due to typically increased *α*. Nevertheless, *k* can always be accurately determined at the $\omega \gamma \to 0$ limit in Fig. 2b.

It should be noted that similar devices were fabricated by Marconnet *et al*.^12^ to measure the *k* values of a nanoladder with DC self-heating. However, their nanoladders were only patterned in the middle 10-μm-long region of a suspended nanobridge, adding complexity to the data analysis. To simplify, this work patterned nanopores across a whole suspended film. The introduction of AC heating not only improved the measurement accuracy but also allowed investigation of the specific heat to justify the hypothesized phononic effects. When compared with measurements loading the thin-film samples onto a suspended microdevice^10,11,21^, the current measurement device integrated the film and the temperature sensor (i.e., metal coating) and thus eliminated the critical thermal contact between the film and the microdevice. The sample distortion and possible damage during the transfer were also avoided. For all samples, the AC and DC measurements were compared for the measured *k* values and the divergence was typically within 5%. In estimation, the influence of radiation loss along the sample was below 1‰.

### Estimating ***k*** for the metallic coating

In data analysis, the effective thermal conductance of the measured film also includes the contribution from the metal layer. The thermal conductance and thus ***k*** of the nanoporous Si film can be obtained by subtracting the metal layer contribution. For the in-plane thermal conductance *G*_*m*_ of the metal layer, the Wiedemann–Franz law suggests $k\approx {k}_{E}=L\sigma T$ and thus$\,{G}_{m}=LT/R$, in which *R* is the electrical resistance of the metal layer.

**Figure 3:**
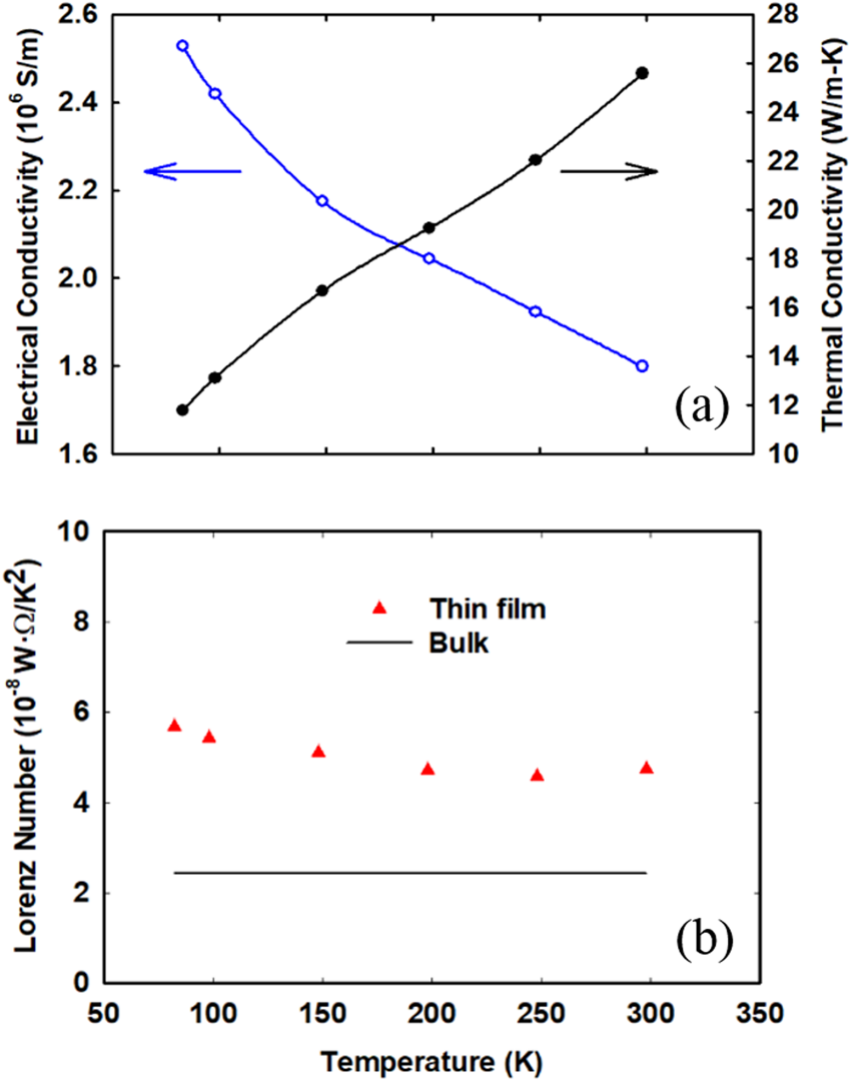
Temperature-dependent (**a**) *k* and *σ*, and (**b**) Lorenz numbers for the Cr/Pt film.

In many studies, the Lorenz number *L* is assigned the Sommerfeld value *L*_0_ = 2.44 × 10^−8^ WΩ/K^2^ for bulk metals^38^. For polycrystalline metallic films prepared by deposition, however, breakdown of the Wiedemann-Franz law is found when some heat carried by charge carriers can be transferred across a grain boundary via phonons though these charge carriers are blocked by the grain boundary. To improve the accuracy of data analysis, *k* has been measured with the 3*ω* technique for a suspended Cr/Pt film that is deposited in the same condition as the metallic coating for nanoporous Si films. Figure 3a shows the measured *k* and *σ*, where the temperature-dependent *L* = $k/\sigma T$ is further presented in Fig. 3b. The computed *L* is lower than *L* ≈ 7 × 10^−8^ WΩ/K^2^ for a 37-nm-thick Au film^39^ but much higher than the Sommerfeld value ${L}_{0}$ = 2.44 × 10^−8^ WΩ/K^2^ for bulk metals. For all temperature-dependent measurements, ${G}_{m}$ of the Cr/Pt coating is within 2.6–26% of the total thermal conductance ${G}_{Total}$ for the bilayer film. The same Lorenz number is approximated for the FIB-patterned films and the estimated ${G}_{m}$ only accounts for 5–13% of ${G}_{Total}$. In this case, further variation in *L* has negligible influence on the extracted *k* values of these nanoporous Si films.

### Electron microscopy studies for pore-edge defects

**Figure 4:**
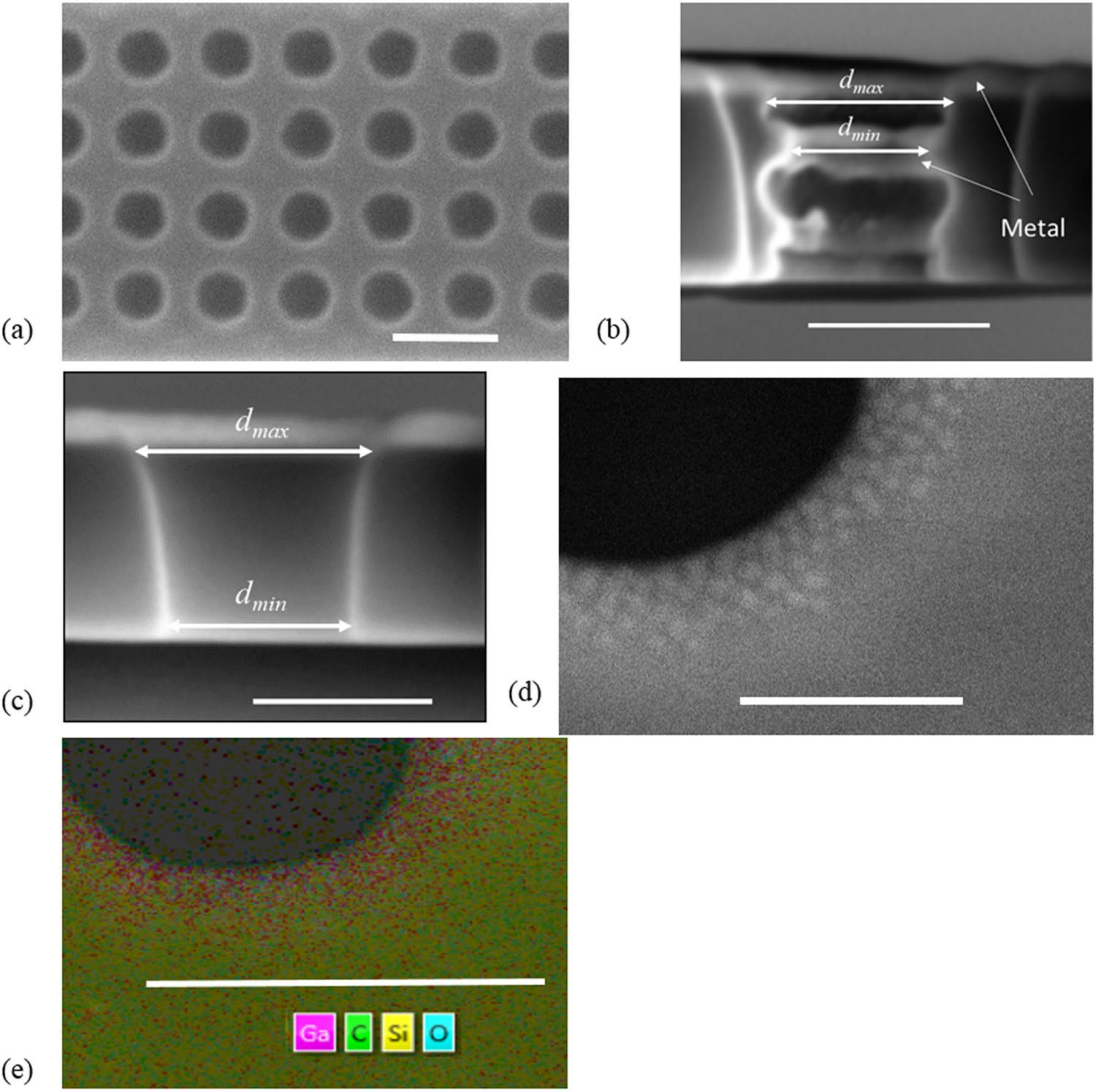
(**a**) Top-view SEM image of a suspended Si film with 200-nm-diameter nanopores. (**b**) Cross-sectional SEM image of a 200-nm-diameter nanopore drilled by DRIE. (**c**) Cross-sectional SEM image of a 200-nm-diameter nanopore drilled by a FIB. (**d**) Dark-field TEM image of a 70-nm-thick Si film with a 200-nm-diameter pore drilled by a FIB. (**e**) Element mapping with EDX for a 250-nm-diameter pore drilled by a FIB on the 70-nm-thick Si film. Scale bars are 500, 250, 200, 100 and 250 nm from (**a**) to (**e**), respectively.

The pore-edge roughness was examined with a SEM^16,17^ for a suspended nanoporous thin film before the metal coating was deposited (Fig. 4a). Such rough pore edges were typically amorphous and often oxidized. In an early study, it was hypothesized that the effective pore diameter should be expanded by the ~200 nm surface roughness of micro-pores drill by DRIE^18^. For DRIE-drilled samples in this study, the width ${\boldsymbol{w}}$ of amorphous edges was roughly 13, 25, 45, and 40 nm for pore sizes of 50(94), 100, 200, and 300 nm, respectively. Representative films after metal deposition were also cut with a FIB and then checked with a SEM to reveal the wavy sidewalls, where *w* determined by the top-view SEM was roughly (*d*_*max*_ − *d*_*min*_)/2 in Fig. 4b. Here *d*_*min*_ was the pore diameter in Fig. 4a. In addition, some metal deposition was also found on the sidewall.

For FIB-drilled samples, the width of the amorphous edge was ~50 nm for both samples from the top-view SEM images but the edge of amorphous region became less clearly defined. Cross-sectional SEM images further showed tapered sidewalls, with $({d}_{max}-{d}_{min})/2\approx 50\,nm$ in Fig. 4c. In the literature, the damage induced by FIB drilling may include an amorphous surface layer of ~10 nm thickness, Ga ion implantation, lattice defects (vacancies, interstitials, and dislocations), and large atom displacement within the collision cascade that extends tens of nanometers from the targeted surface^36^. Due to its exposure under the ion beam, the tapered sidewall was anticipated to be highly amorphous within the ~50 nm edge. Because the current 220 nm film thickness was too thick for TEM studies, the pore-edge defects were cross-checked with a TEM using a 70-nm-thick film drilled with a FIB. For 200-nm-diameter pores, the amorphous region was 50–70 nm wide (Fig. 4d). Further check with energy dispersive x-ray spectroscopy (EDX) identified Ga ion implantation both within and outside the amorphous edge (Fig. 4e).

### Phonon MC simulations

For lightly doped Si films, the electronic contribution to *k* is neglected so that $k\approx {k}_{L}$ is assumed. The exact *k*_*L*_ value is predicted with phonon MC simulations that track the movement and scattering of individual phonons. A single period of the nanoporous film is used as the computational domain, using a boundary condition based on the periodic heat flux with a constant virtual wall temperature for periodic structures^22^. When the extracted temperature profile converges, *k*_*L*_ is computed from the temperature difference and periodic heat flux across the simulated structure. The exact geometry of the nanoporous structure and energy-dependent phonon MFPs can be fully incorporated into such simulations^23^. In calibration with bulk materials, the accuracy of these phonon MC simulations is <4% in general^22^. To improve the computational efficiency, a variance-reduced MC technique developed by Péraud and Hadjiconstantinou has been employed^40^. This technique can dramatically improve the computational efficiency by simulating “useful” phonons only corresponding to the distribution function deviation from a reference equilibrium distribution. In all simulations, phonon dispersion modification due to coherent phonon transport is not considered because the nanoporous pattern was much larger than the 1–10 nm phonon wavelengths for Si at 300 K^27,28^.

**Figure 5:**
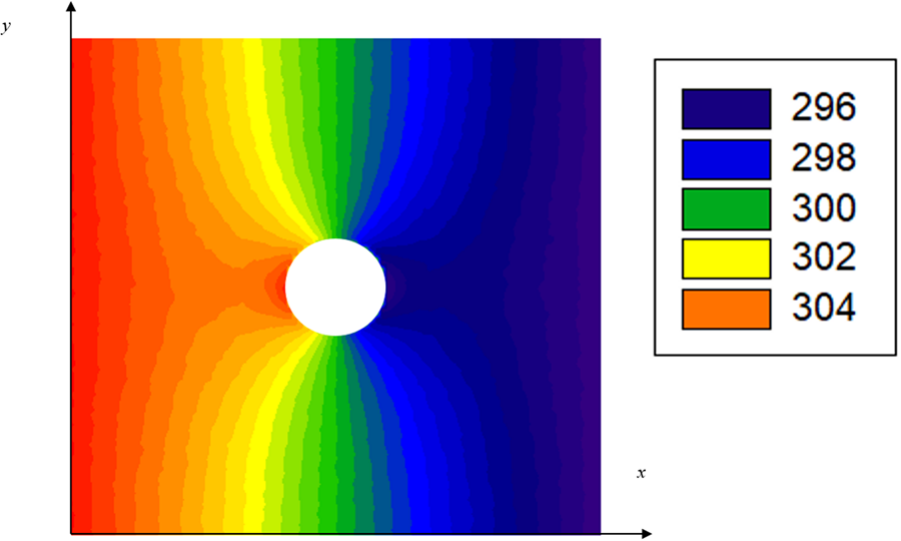
Typical top-view temperature profile from a phonon MC simulation for one period (50 nm × 50 nm) of a nanoporous film. A periodic heat flux is applied along the positive *x* direction. Temperature is in Kelvin.

In previous phonon transport studies on nanoporous Si films, phonon MFP sampling and ray tracing with the MC technique were also used^12,19,27,41^. These techniques tracked the transport of a single phonon and statistically obtained the reduced MFP of this phonon due to boundary scattering. In the summation of *k*_*L*_, the contribution of each phonon was modified for its suppressed phonon MFP. Without computing the exact temperature profile, such MFP-based calculations did not consider details such as the phonon energy reset after phonon-phonon scattering and phonon “necking effects” to distort the temperature distribution around the pores (Fig. 5). In contrast, the phonon MC simulations in this work fully considered all phonon scattering processes and the exact temperature distribution, which was anticipated to be more accurate for *k*_*L*_ predictions of complicated three-dimensional nanostructures.

In phonon MC simulations, the top and bottom surfaces of a nanoporous film were assumed to only diffusively scatter phonons, which provided the lower bound of *k*_*L*_. In addition, the rough and highly amorphous edges around each pore would introduce non-propagating, diffusive lattice-vibration modes to largely suppress heat transport^32^. To simplify, the pore diameter was expanded by the width of the observed amorphous edge so that ${d}_{max}$ in Fig. 4b and c were used as the effective pore diameter. A similar treatment was proposed by Ravichandran and Minnich^28^.

**Figure 6:**
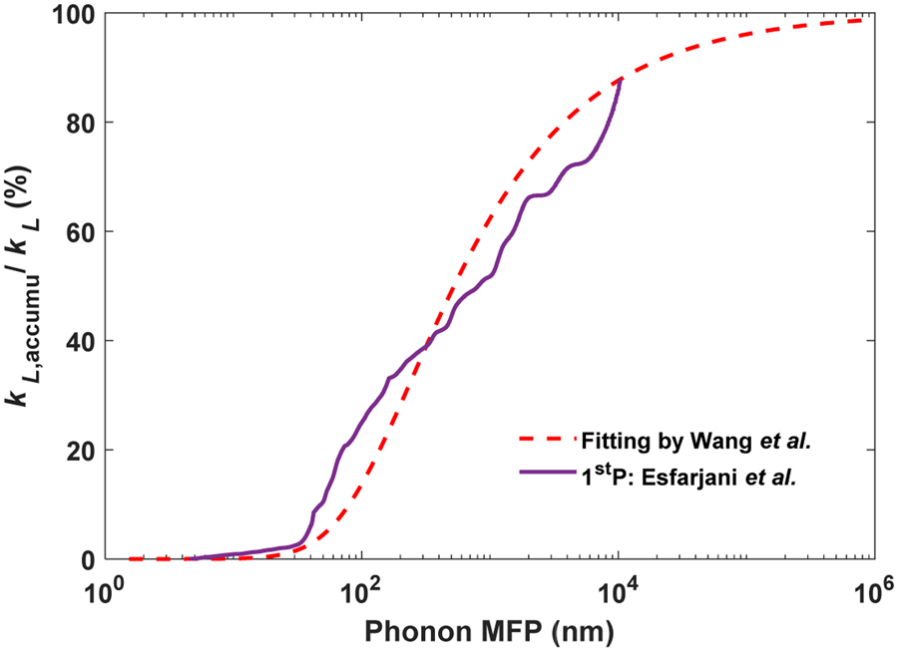
Room-temperature accumulated *k*_*L*_ from different phonon parameters by first-principles (1^st^ P) and data fitting.

As the input parameter, the bulk phonon MFPs are fitted by Wang *et al*. for bulk-Si *k*_*L*_^42^ (See Supplementary Information). An isotropic phonon dispersion and three identical sine-shape acoustic branches are assumed here. Despite these simplifications, the spectral *k*_*L*_ at 300 K agrees well with accurate first-principles predictions by Esfarjani *et al*.^43^ (Fig. 6). The first-principles calculations are supported by measurements on bulk Si for spectral *k*_*L*_^44^. With similar spectral *k*_*L*_, the two bulk phonon MFP distributions are anticipated to yield very close *k*_*L*_ values for the same nanostructure with phonon boundary scattering^45^. At room temperature, the simulation results using first-principles or fitted phonon parameters diverge within 3% for 220-nm-thick nanoporous films with $\Phi$=19.6% and a pitch of 200–600 nm. Because temperature-dependent phonon MFPs are not available for calculations by Esfarjani *et al*., the fitted bulk phonon MFPs and simplified phonon dispersion are used here.

### Thermal measurement results

Representative ~100 nm nanoporous patterns were investigated for their impact on *k*. As the reference, a solid film was measured and its room-temperature *k*≈86 W/m·K agreed well with *k* ≈ 85 W/m·K in previous studies^46^. For each nanoporous pattern, two to four samples were measured for films drilled by DRIE. At room temperature, the standard deviation of *k* was within 2.3–5.1% of the average *k* values for each pattern, which suggested high repeatability of our measurements. In addition, two FIB-drilled samples were measured and showed *k* lower than that for films drilled by DRIE. The detailed uncertainty analysis of both *k* and *C* measurements was provided in Supplementary Information, with <5% uncertainty estimated for all measurements on nanoporous Si films.

**Figure 7:**
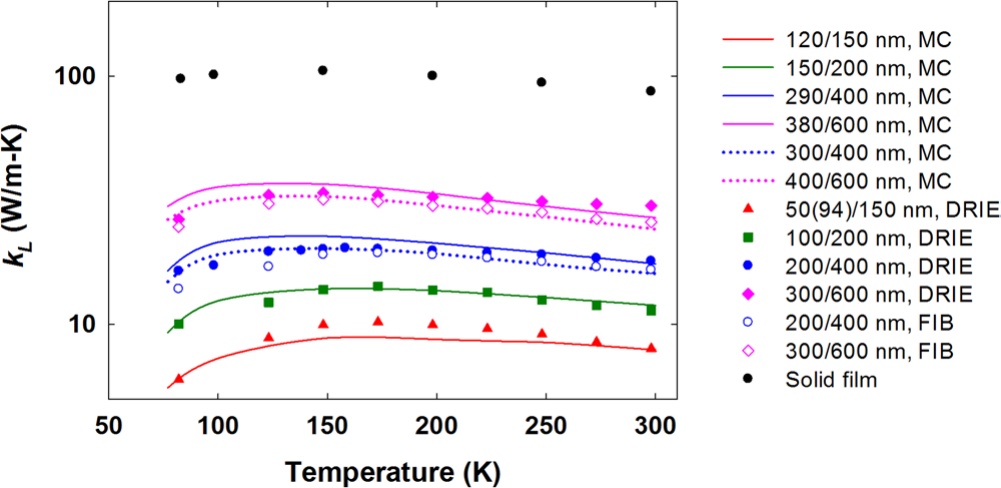
Temperature-dependent *k*_*L*_ of the solid and nanoporous Si thin film (symbols), in comparison to predictions by MC simulations (lines) using an effective pore diameter indicated in the legend. The diameter/pitch combinations are given in the legend. The colour of lines matches the corresponding measurement data. The solid lines and filled symbols are for DRIE samples, while the dashed lines and hollow symbols are for two FIB samples.

Figure 7 compares the measurements and simulations for temperature-dependent $k\approx {k}_{L}$ of all nanoporous thin films. The effective pore diameters are taken as ${d}_{max}$ shown in Fig. 4b, which is 120, 150, 290, and 380 nm for the 50(94), 100, 200, and 300 nm pore diameters in DRIE-drilled films, respectively. For FIB-drilled films, the effective diameters for 200 and 300 nm nominal pore diameters are increased by 100 nm, as ${d}_{max}$ in Fig. 4c. The Ga ion implantation during FIB cutting can further lower *k* with stronger point-defect scattering of phonons. Such effects are not considered here to simplify the analysis. In general, the measurement data agree well with predictions by phonon MC simulations using effective pore diameters.

**Figure 8:**
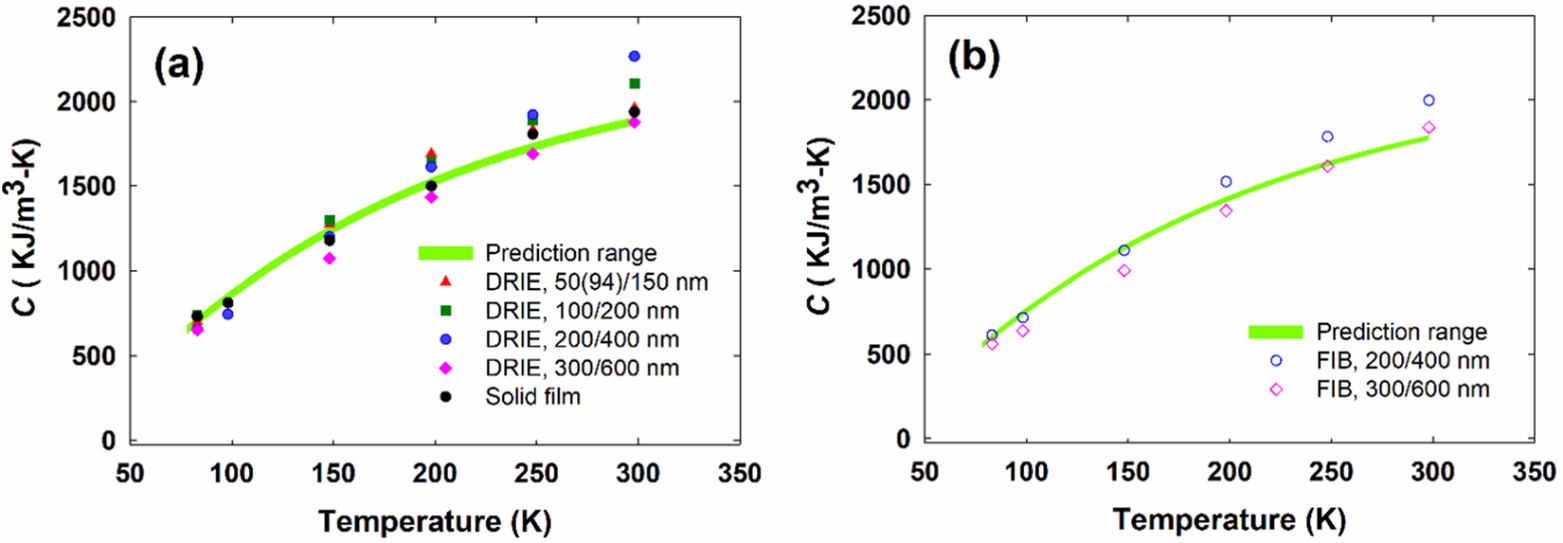
Temperature-dependent solid *C* of bilayer films drilled by (**a**) DRIE and (**b**) a FIB, in comparison to the prediction using bulk *C* values for metals and Si.

As one highlight of this work, Fig. 8a presents the corresponding solid volumetric specific heat *C* for all bilayer films patterned with DRIE. For nanoporous films, all measured *C* values are divided by $(1-\Phi )$. In estimation, half of the volume between two cylinders with diameters of ${d}_{min}$ and ${d}_{max}$ (Fig. 4b and c) is assumed to be empty and is added to $\Phi$ as the correction (see Supplementary Information). In comparison, *C* is also computed using the bulk specific heat for each material layer and their thicknesses. In SEM examination of the FIB-cut cross section, ±5 nm thickness uncertainties have been found in the Si and metallic layers. The corresponding range of the prediction is indicated by the green band. Good agreement can be observed between the solid film (filled black circle) and the prediction. Some divergence of solid *C* is found for nanoporous films, which can be attributed to the inaccuracy in $\Phi$ and additional pore-edge defects. In general, the solid *C* of nanoporous Si films still follow the trend for a solid film. Similar comparison is also displayed for the two nanoporous films drilled by a FIB (Fig. 8b). The bulk-like *C* values for nanoporous films suggest negligible phonon dispersion modification and thus weak phononic effects. Our conclusion here contradicts previously claimed phononic effects for a Si film with pitches of 500–800 nm^24^.

## Discussion

Numerous studies have been carried out on nanoporous Si thin films to understand how to manipulate phonon transport for various important applications^47^. Different from the predicted phonon behaviour in nanoporous thin films, measurements on real samples are often affected by the defects introduced by nanofabrication, such as amorphization and oxidation on pore edges. To observe phononic effects, ultra-fine periodic nanofeatures and/or cryogenic temperatures are required^16,19,20^. In addition, the negative impact of amorphous pore edges should also be addressed when the wave nature of lattice vibrations is employed for applications using nanostructures. In this aspect, DRIE drill can introduce wavy sidewalls with amorphous edges so that *k* can be further reduced for DRIE-patterned films^11,18–20^. For FIB-drilled samples, even more defects are expected and special treatment must be taken to reduce the pore-edge damage^21^.

In phonon modeling, *k* of a nanoporous film can be predicted by accurately incorporating the phonon size effects. For two-dimensional nanoporous thin films, the mean beam length *L* of the structure can be used as the characteristic length to modify the phonon bulk MFP ${{\rm{\Lambda }}}_{Bulk}$^23^, similar to the phonon MFP modification by the diameter of a nanowire^38^. Widely used for radiation, the mean beam length is given as $L=4V/S$, where the solid-region volume *V* and pore surface area *S* depend on the period and pore diameter. At low temperatures, the bulk phonon MFPs are much longer than *L* so that *L* can be approximated as the effective phonon MFP. For three-dimensional films, the film thickness can be further added to *L* using the Matthiessen’s rule, as shown for cross-plane *k* studies^31^. The above treatment combines different geometry parameters into a single parameter to modify the bulk phonon MFPs. On the other hand, the specific heat of our measured nanoporous films only depends on the porosity and pore-edge defects. The structure sizes have no influence when phononic effects are negligible.

Without the pore drilling processes, nanoporous films can be directly grown by metalorganic chemical vapor deposition with SiO_2_ pillar as masks^31^, which can be removed with hydrofluoric acid later. This can eliminate the pore-edge damage by RIE, DRIE, or FIB. When phonon coherence is preferred, such nanostructures may be used to better conserve the wave effects. The surface defects can also be critical to other periodic structures, such as silicon nanowire cage structures^48^.

## Conclusions

In this work, systematic thermal studies have been carried out on nanoporous Si thin films drilled with DRIE and a FIB. For nanoporous patterns with ~100 nm feature sizes, the specific heat measurements do not suggest significantly changed phonon dispersions. Compared to some previous experimental studies, our measured in-plane *k* values of the nanoporous Si films agree with three-dimensional phonon MC simulations when the pore expansion due to amorphous pore edges is considered. The direct comparison between DRIE- and FIB-drilled films indicates the impact of increased pore-edge defects on *k* reduction. The results presented here provide important guidance for “phonon engineering” within nanostructures fabricated by different techniques, such as nanoporous graphene patterned with RIE or FIB^49–51^.

## Electronic supplementary material

Supplementary Information
