# Supplementary material for: Thermal Studies of Nanoporous Si Films with Pitches on the Order of 100 nm —Comparison between Different Pore-Drilling Techniques
Source: Sci Rep. 2018 Jun 13;8:9056. doi: 10.1038/s41598-018-26872-w (PMC5998148; doi:10.1038/s41598-018-26872-w)
Supplement: Supplementary file 1 — Supplementary Information [file 41598_2018_26872_MOESM1_ESM.docx]

**Supplementary Information**

Thermal Studies of Nanoporous Si Films with Pitches on the Order of 100 nm — Comparison between Different Pore-Drilling Techniques

Qing Hao, Dongchao Xu, Hongbo Zhao, Yue Xiao and Fabian Javier Medina

Aerospace & Mechanical Engineering, University of Arizona, 1130 N Mountain Ave, Tucson, AZ 85721, U.S.A.

**1. 3*ω* measurements and uncertainties of** $\boldsymbol{k}$ **and *C***

In 3*ω* measurements of a suspended sample, the frequency-dependent voltage $V_{3\omega}$ and its phase $\theta$ are given as^1^

$tan\theta\approx2\omega\gamma$, (S1)

$V_{3\omega}\approx\frac{4I^{3}RR^{'}}{\pi^{4}\sqrt{1+\left( 2\omega\gamma\right)^{2}}}\frac{l}{kS}$, (S2)

in which *V*_3_*_ω_* and current $I$ are both in root-mean-square (RMS) values, $l$ is the sample length, $S$ is the cross-section area, $R$ is the electrical resistance and $R^{'}=dR/dT$. The time constant $\gamma$ for the length-direction heat spreading along the sample is

$\gamma=l^{2}/{(\pi}^{2}\alpha)$, (S3)

with *α* as the thermal diffusivity. In data analysis, $\gamma$ is given by fitting $\tan\theta\approx2\omega\gamma$ (Fig. 2a). Fitting the $V_{3\omega}\sim1/k\sqrt{1+\left( 2\omega\gamma\right)^{2}}$ curve further yields $k$ (Fig. 2b). The specific heat per unit volume $C$ is then given as

$C\approx\pi^{2}k\gamma/l^{2}$, (S4)

which can be divided by $1-\Phi$ to find the specific heat for the solid counterpart of a porous film.

When the suspended sample consists of a Si film and a metallic coating layer, the thermal resistance of the bilayer film, $l/(kS)$ in Eq. (S2), becomes the equivalent thermal resistance of the bilayer film. The fitted $k$ can be viewed as the effective thermal conductivity $k_{eff}$ for the whole bilayer film. This effective $k$ in Eq. (S2) is then used in Eq. (S4) to compute $C$ for the bilayer film.

In measurements, the constant AC current is provided by the Keithley 6221 DC/AC current source, with 1% error. The 1*ω* signal across the film is subtracted using a common differential amplifier circuit with a reference resistor serially connected with the measured film. The 3*ω* signal is collected by a SR830 lock-in amplifier (Stanford Research Systems). The gain accuracy of the lock-in is considered as 1%. The electrical resistance of the film, which is mainly contributed by the metal layer, is measured as a function of temperature to extract $R^{'}$ in Eq. (S2). The temperature coefficient of resistance of the metal layer, $R^{'}$/*R*_300K_, is calibrated for each sample and used in the thermal analysis. In addition, the temperature of the cryogenic-chamber is also read from the $R$ variation of each sample when a weak sensing current is used for $R$ measurements.

For all temperature-dependent measurements, the maximum AC frequency is up to ~20 kHz. The RMS value of the heating current is fixed at 0.25–0.5 mA. For a solid film, the current is increased to 1 mA at low temperatures to enhance the signal. In all cases, the average temperature rise is 2–5 K along the film. The relatively large film length is particularly selected to increase the time constant $\gamma\sim L^{2}$ for 3*ω* measurements. When the slope of the $tan(\theta)-\omega$ curve is fitted to extract $\gamma$, the small relative phase error (<0.01° for SR830) ensures accurate $\gamma$ measurements even for a solid thin film with the lowest $\gamma$ value.

The uncertainty of the time constant $\gamma$ in Eq. (S1) is

$\mu_{\gamma}=\frac{dtan\emptyset}{d\emptyset}\frac{\mu_{\emptyset}}{2\omega}=\frac{\mu_{\emptyset}}{2\omega\cos^{2} \emptyset}$, (S5)

where the uncertainty of the phase angle is $\mu_{\emptyset}={0.01}^{^{\circ}}$ and $\mu_{\gamma}$ is estimated at the maximum angular frequency $\omega$.

Using Eq. (S2) and fitted $\gamma$, the thermal conductance *G* of the bilayer film is

$G=k_{eff}S/l\approx\frac{4I^{3}RR^{'}}{\pi^{4}\sqrt{1+\left( 2\omega\gamma\right)^{2}}}\frac{1}{V_{3\omega}}=\frac{4I^{2}V_{1\omega}R^{'}}{\pi^{4}\sqrt{1+\left( 2\omega\gamma\right)^{2}}}\frac{1}{V_{3\omega}}$, (S6)

in which the RMS value of 1*ω* voltage $V_{1\omega}$ is used to evaluate the electrical resistance $R$ of a thin film at a given temperature. The uncertainty of $G$ is

$\mu_{G}=\left[ \left( \frac{\partial G}{\partial I} \right)^{2}\left( \mu_{I} \right)^{2}+\left( \frac{\partial G}{\partial V_{1\omega}} \right)^{2}\left( \mu_{V_{1\omega}} \right)^{2}+\left( \frac{\partial G}{\partial V_{3\omega}} \right)^{2}\left( \mu_{V_{3\omega}} \right)^{2}+ \left( \frac{\partial G}{\partial\gamma} \right)^{2}\left( \mu_{\gamma} \right)^{2} \right]^{1/2}$. (S7)

The thermal conductance and thus thermal conductivity of the solid or nanoporous Si film can be obtained by subtracting the metal layer contribution. For the in-plane thermal conductance $G_{m}$ of the metal layer, the Wiedemann–Franz law suggests $k\approx k_{E}=L\sigma T$ and thus $G_{m}$ = $LT/R$, in which $R$ is the electrical resistance of the metal layer, and $L$ is the Lorenz number. The thermal conductivity $k_{Si}$ of the Si film is

$k_{Si}=\frac{\left( G-G_{m} \right)l}{S_{Si}}= \frac{\left( G-LT/R \right)l}{S_{Si}}=\frac{Gl}{Wt_{Si}}-\frac{LTIl}{V_{1\omega}Wt_{Si}}$, (S8)

where $W$ and $t$ are the width and thickness of a film, respectively. The subscript “Si” and “m” indicates Si and metallic coating, respectively. Here the error in the length $l$ =20 µm and width *W* =2 µm of a suspended film can be neglected, whereas the Si film thickness $t_{Si}$ still has 5 nm uncertainty for its 220 nm thickness. The overall uncertainty of $k_{Si}$ is

$\mu_{k_{Si}}=\left[ \left( \frac{\partial k_{Si}}{\partial G} \right)^{2}\left( \mu_{G} \right)^{2}+\left( \frac{\partial k_{Si}}{\partial I} \right)^{2}\left( \mu_{I} \right)^{2}+\left( \frac{\partial k_{Si}}{\partial V_{1\omega}} \right)^{2}\left( \mu_{V_{1\omega}} \right)^{2}+\left( \frac{\partial k_{Si}}{\partial t_{Si}} \right)^{2}\left( t_{Si} \right)^{2} \right]^{1/2}$. (S9)

For a solid Si film, the uncertainty is $\mu_{k_{Si}}\leq3.5 W/(m\cdot K)$ and the relative error is within 3.5% at all temperatures. For nanoporous films, the relative error is less than 4.5% in general.

Using Eq. (S4), the uncertainty of the volumetric specific heat of the bilayer film is

$u_{C}=\sqrt{\left( \frac{\partial C}{\partial\gamma} \right)^{2}\left( \mu_{\gamma} \right)^{2}+\left( \frac{\partial C}{\partial k} \right)^{2}\left( \mu_{k} \right)^{2}}$. (S10)

For a solid Si film, the uncertainty is $u_{C}\leq80 kJ/(m^{3}\cdot K)$ and the relative error is within 11% in the whole temperature range. For nanoporous films, the relative error is within 5% at all temperatures.

**2. Porosity calculations for nanoporous films**

Consider a nanopore with an outer diameter $d_{max}$ (radius $r_{max}$) and inner diameter $d_{min}$ (radius $r_{min}$) for the rough pore edge. Assuming the volume is half filled from $d_{min}$ to $d_{max}$ around each nanopore, the porosity is estimated as

$\phi=\frac{\pi r_{min}^{2}+(\pi r_{max}^{2}-\pi r_{min}^{2})/2}{p^{2}}$, (S11)

with $p$ as the period of the structure. All parameters are listed in Table S1. Note all diameter values have <5 nm uncertainties in the measurement using a scanning electron microscope (SEM).

Table S1. Geometry parameters for different samples.

| Drilling technique | Period (nm) | *d_max_* (nm) | *d_min_*(nm) | Corrected *Φ* |
| --- | --- | --- | --- | --- |
| Deep reactive ion etching | 150 | 120 | 94 (over-etched from 50 nm) | 41% |
|  | 200 | 150 | 100 | 32% |
|  | 400 | 290 | 200 | 30% |
|  | 600 | 380 | 300 | 26% |
| Focused ion beam | 400 | 300 | 200 | 32% |
|  | 600 | 400 | 300 | 27% |

**3. Input parameters for phonon MC simulations**

In all phonon MC simulations, an isotropic phonon dispersion is assumed. Currently, considering the full phonon dispersion is still not feasible for phonon MC simulations due to the dramatically increased computational load. To simplify, three identical acoustic phonon branches are adopted and a sine-shaped phonon dispersion (Born-von Karman dispersion) is used. In this dispersion, the phonon angular frequency $\omega$ is expressed as

$\omega=\omega_{\max}\sin\left( \pi q/2q_{0} \right)$, (S12)

in which $\omega_{\max}$ and $q_{0}$ are the maximum phonon angular frequency $\omega$ and wavevector $q$, respectively. Here $q_{0}$ can be calculated using $q_{0}=\frac{\pi}{a_{D}}=\left( 6\pi^{2}N \right)^{1/3}$, with $a_{D}$ as the equivalent atomic distance and $N$ as the volumetric density of primitive cells. The maximum angular frequency can be calculated from $a_{D}$ as $\omega_{\max}=\frac{2v_{s}}{a_{D}}$.

Frequency-dependent phonon relaxation time for bulk Si, denoted as $\tau\left( \omega\right)$, is given as^2^

$\frac{1}{\tau\left( \omega\right)}=A\omega^{4}+B_{1}\omega^{2}T\exp\left( -\frac{B_{2}}{T} \right)$, (S13)

where the first term on the right-hand side accounts for the impurity-phonon scattering, and the second term is for the Umklapp scattering. All employed parameters are given in Table S2.

Table S2. Parameters used by Wang *et al*.^2^

| $a_{D}$  (Å) | $\omega_{\max}$  (rad/s) | $q_{0}$  (m^-1^) | $A$  (s^3^) | $B_{1}$  (s/K) | $B_{2}$  (K) |
| --- | --- | --- | --- | --- | --- |
| 2.75 | $4.41 \times{10}^{13}$ | $1.14 \times{10}^{10}$ | $1.69 \times{10}^{-45}$ | $1.53 \times{10}^{-19}$ | $140$ |

References:

1. L. Lu, W. Yi and D. Zhang, *Review of Scientific Instruments*, 2001, **72**, 2996-3003.

2. Z. Wang, J. E. Alaniz, W. Jang, J. E. Garay and C. Dames, *Nano Letters*, 2011, **11**, 2206-2213.
